# Effects of trunk neuromuscular electrical stimulation on the motor circuits of able-bodied individuals

**DOI:** 10.1007/s00221-023-06585-x

**Published:** 2023-03-14

**Authors:** Atsushi Sasaki, Na Cao, Akiko Yuasa, Milos R. Popovic, Kimitaka Nakazawa, Matija Milosevic

**Affiliations:** 1 Graduate School of Engineering Science, Department of Mechanical Science and Bioengineering, Osaka University, 1-3 Machikaneyama, Toyonaka, Osaka 560-8531 Japan; 2 Department of Life Sciences, Graduate School of Arts and Sciences, The University of Tokyo, 3-8-1 Komaba, Meguro-Ku, Tokyo, 153-8902 Japan; 3 Japan Society for the Promotion of Science, 5-3-1 Kojimachi, Chiyoda-Ku, Tokyo, 102-0083 Japan; 4 Department of Rehabilitation Medicine I, Fujita Health University School of Medicine, 1-98 Dengakugakubo, Kutsukake-Cho, Toyoake, Aichi 470-1192 Japan; 5 Institute of Biomaterials and Biomedical Engineering, University of Toronto, 164 College Street, Toronto, ON M5S 3G9 Canada; 6 KITE, Toronto Rehabilitation Institute - University Health Network, 550 University Ave., Toronto, ON M5G 2A2 Canada; 7 CRANIA, University Health Network, 550 University Ave., Toronto, ON M5G 2A2 Canada

**Keywords:** Neuromuscular electrical stimulation, Trunk, Corticospinal tract, Neuromodulation

## Abstract

Upper- and lower-limb neuromuscular electrical stimulation (NMES) is known to modulate the excitability of the neural motor circuits. However, it remains unclear whether short-duration trunk muscle NMES could achieve similar neuromodulation effects. We assessed motor evoked potentials (MEPs) elicited through transcranial magnetic stimulation of the primary motor cortex representation of the trunk extensor muscles to evaluate corticospinal excitability. Moreover, cervicomedullary motor evoked potentials (CMEPs) were assessed through cervicomedullary junction magnetic stimulation to evaluate subcortical excitability. Twelve able-bodied individuals participated in the MEP study, and another twelve in the CMEP study. During the interventions, NMES was applied bilaterally to activate the erector spinae muscle and produce intermittent contractions (20 s ON/20 s OFF) for a total of 20 min while participants remained seated. Assessments were performed: (i) before; (ii) during (in brief periods when NMES was OFF); and (iii) immediately after the interventions to compare MEP or CMEP excitability. Our results showed that MEP responses were not affected by trunk NMES, while CMEP responses were facilitated for approximately 8 min during the intervention, and returned to baseline before the end of the 20 min stimulating period. Our findings therefore suggest that short-duration NMES of the trunk extensor muscles likely does not affect the corticospinal excitability, but it has a potential to facilitate subcortical neural circuits immediately after starting the intervention. These findings indicate that short-duration application of NEMS may be helpful in rehabilitation to enhance neuromodulation of the trunk subcortical neural motor circuits.

## Introduction

The human spine is inherently unstable and the trunk muscles surrounding the spine play an important role in maintaining trunk stability and upright posture (Milosevic et al. 2017). Since it is known that neurological impairments such as spinal cord injury can affect the control of trunk muscles, which could also cause sitting balance impairments (Milosevic et al. 2015a, 2017), development of an effective neuromodulation technique for activating the trunk neural circuits could provide implications for rehabilitation to improve trunk function. Previous studies with able-bodied participants have shown that neuromuscular electrical stimulation (NMES) could generate muscle contractions by depolarising the axons under the stimulating electrodes, including both the efferent (motor) and the afferent (sensory) fibers (Bergquist et al. 2011; Carson and Buick 2021). Depolarization of the motor axons during NMES can induce muscle contractions without the central (voluntary) commands in prosthetic applications aiming to produce functional movements. At the same time, sensory fiber activation provides afferent feedback to the central nervous system which can contribute to the enhanced excitability (i.e., neuromodulation) and to the restoration of sensorimotor function after neurological injuries (Bergquist et al. 2011; Carson and Buick 2021). Application of NMES has been successfully used to generate trunk muscle contractions, as well as to improve spinal alignment, sitting posture, trunk stability, and reaching ability in individuals with spinal cord injury (Kukke and Triolo 2004; Triolo et al. 2009). Moreover, NMES applications were used to stabilize the trunk during wheelchair perturbations, which can also prevent falls and injuries (Patel et al. 2017; Armstrong et al. 2018). While prosthetic NMES application has been applied successfully in clinical settings, it remains unclear how short-duration (< 20 min) NMES of the trunk muscles affects the intact central nervous system excitability. Therefore, understanding the neurophysiological effects of NMES will help uncover potential NMES applications for neuromodulation which would contribute to an effective rehabilitation approach. In our current study, we first focused on able-bodied participants as an initial step toward gathering fundamental evidence before translating these findings to the neurological injury population in the future.

Numerous previous studies have investigated neuromodulation applications of upper- and lower-limb NMES in able-bodied individuals. These studies suggests that 20 to 40 min of NMES could increase upper-limb (Barsi et al. 2008; Mang et al. 2011) and lower-limb (Knash et al. 2003; Khaslavskaia and Sinkjaer 2005; Mang et al. 2010, 2011; Thompson et al. 2011) corticospinal excitability immediately after the intervention, and that the effects could last anywhere between 30 and 60 min after the intervention is completed (e.g., Knash et al. 2003; Khaslavskaia and Sinkjaer 2005). Moreover, the effects of NMES on spinal excitability have also been evaluated primarily in the lower-limb ankle muscle group (i.e., tibialis anterior and soleus muscles) (Kitago et al. 2004; Thompson et al. 2011; Jimenez et al. 2018; Milosevic et al. 2019). Specifically, Kitago and colleagues (Kitago et al. 2004) reported that 10 min of intermittent (i.e., ON/OFF) NMES application could facilitate spinal excitability of the soleus muscles and that the after-effects could last between 4 and 16 min after the intervention completion (Kitago et al. 2004). On the other hand, reduced spinal excitability of the soleus muscles was also reported immediately after continuous NMES interventions (Jimenez et al. 2018; Milosevic et al. 2019), with after-effects persisting for at least 15 min (Milosevic et al. 2019). Conversely, it was shown that 30 min of NMES does not affect spinal excitability of the tibialis anterior muscles (Thompson et al. 2011). Therefore, NMES could facilitate corticospinal excitability of the upper- and lower-limb muscles with prolonged after-effects beyond the stimulation period, while spinal excitability of the lower-limb muscles could be facilitated, inhibited, or even remain unaffected depending on which muscles are stimulated and/or the stimulation pattern (i.e., continuous or intermittent NMES).

A recent report by Elgueta-Cancino and colleagues suggested that trunk NMES does not affect intracortical circuits as well as the corticospinal excitability after short-duration stimulation intervention (Elgueta-Cancino et al. 2019). However, they investigated the effects of trunk NMES only after the intervention was completed (Elgueta-Cancino et al. 2019). Therefore, investigating central nervous system effects during the intervention could provide further insights into NMES effects and help to determine intervention duration to maximize neuromodulation effects. Moreover, since trunk muscles have stronger projections from the subcortical circuits (i.e., the brainstem, vestibular networks, and the spinal cord), rather than the motor cortex (Cottingham et al. 1988; Zedka et al. 2004; Galea et al. 2010), it is also more likely that subcortical motor circuits would be affected by a short-duration trunk NMES. Recent studies reported that cervicomedullary junction magnetic stimulation (CMS) can reliably assess spinal neural excitability of the trunk muscles (Jean-Charles et al. 2017; Chiou et al. 2018a, b), which are otherwise difficult to evaluate using standard techniques (e.g., H-reflex) due to inaccessibility of the trunk nerves. Therefore, by assessing cervicomedullary motor evoked potentials (CMEPs) elicited through CMS (subcortical excitability), in addition to motor evoked potentials (MEPs) elicited through transcranial magnetic stimulation (TMS) of the primary motor cortex (corticospinal excitability), it is possible to gain a better understanding about the neuromodulation effects of trunk NMES.

The aim of this study was to investigate how NMES of the trunk extensor muscles affects corticospinal excitability during and after a short-duration intervention. We hypothesized that short-duration application of trunk muscle NMES would not affect MEP responses of the trunk muscles as recently shown (Elgueta-Cancino et al. 2019), while CMEP responses would be facilitated as shown in the lower-limb muscles during intermittent application of NMES (Kitago et al. 2004).

## Materials and methods

### Participants

A total of seventeen able-bodied individuals were recruited for the current study. Twelve out of seventeen participated in the MEP session (male; age: 24.9 ± 1.8 years, weight: 70.6 ± 9.2 kg, and height: 174.3 ± 5.7 cm; mean ± SD) and twelve out of seventeen participated in the CMEP session (male; age: 25.5 ± 2.5 years, weight: 68.9 ± 7.9 kg, and height: 173.6 ± 5.0 cm; mean ± SD). It should be noted that seven participants took part in both sessions. The sample size for this study was determined based on a previous study that investigated the effect of trunk NMES on corticospinal excitability (Elgueta-Cancino et al. 2019). None of the participants had a history of neurological and/or musculoskeletal impairments which would prevent their participation (Rossini et al. 2015). All participants gave written informed consent in accordance with the Declaration of Helsinki, which was approved by the local institutional ethics committee of the Graduate School of Arts and Science at The University of Tokyo.

### Experimental protocol

**Fig. 1:**
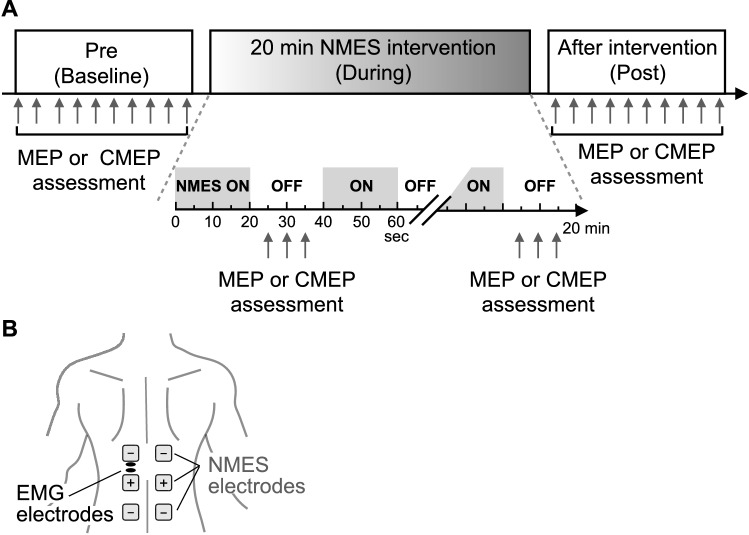
**A** Experimental protocol - Experiment consisted of cervicomedullary evoked potential (CMEP) or motor evoked potential (MEP) sessions. Experimental data were obtained: (i) at baseline (Pre), (ii) during the NMES intervention (During) and (iii) after the intervention (Post). During the intervention, NMES was delivered intermittently (20 s ON/20 s OFF) for a total of 20 min. Assessments included recording CMEP or MEP responses: ten stimuli were delivered in the Pre and Post assessments and three stimuli were delivered for each NMES OFF (20 s) period in the During assessments. **B** Electromyographic (EMG) activities were recorded from the trunk - erector spinae on the 12th thoracic vertebral level. Surface electrodes for neuromuscular electrical stimulation were placed on the upper back and lower back regions (anode: + and cathode: −)

During the experiments, participants were seated comfortably on a chair without a backrest with both arms relaxed on the side of their body and were asked to maintain an upright posture of the trunk. Assessments were performed: (i) at baseline before the intervention (Pre); (ii) during the 20 min intervention in brief periods when NMES was not applied (During); and (iii) immediately after the intervention (Post). Based on previous studies investigating the neurophysiological effect of NMES in upper-limb (Barsi et al. 2008; Mang et al. 2011), lower-limb (Knash et al. 2003; Khaslavskaia and Sinkjaer 2005; Mang et al. 2010, 2011; Thompson et al. 2011) and trunk (Elgueta-Cancino et al. 2019) muscles, a 20-min NMES intervention was investigated to test short-duration neuromodulation effects. NMES was delivered intermittently: 20 s ON/20 s OFF (Mang et al. 2010), and MEP and CMEP responses were assessed in two separate experiments. Ten MEP or CMEP responses were elicited in each of the Pre and Post assessments, while three responses were elicited in each of the NMES OFF periods in the During assessment phase (i.e., each 20 s OFF window; Fig. 1A), consistent with a previous study (Mang et al. 2010), which resulted in a total of 90 MEPs or CMEPs during the 30 ON/OFF sets. Responses were always elicited every 5 s by triggering the stimulator using a custom-written MATLAB program (The MathWorks Inc., Massachusetts, USA).

### Neuromuscular electrical stimulation (NMES)

A constant-current electrical stimulator (Complex Motion II, Compex, Switzerland) was used to apply NMES bilaterally to the upper back and lower back regions of the erector spinae (ES) muscles (Fig. 1B). Specifically, NMES was delivered by applying 300 μs rectangular, biphasic, asymmetric charge balanced stimulating pulses at the frequency of 40 Hz using adhesive electrodes (5 × 5 cm) (Milosevic et al. 2015b; Patel et al. 2017; Elgueta-Cancino et al. 2019). The cathode electrodes were placed bilaterally on the ES muscles approximately at the T11 and L4 vertebral levels, while a common anode was also placed bilaterally on the ES muscles approximately at the L1-L2 vertebral level. The top and bottom cathode electrodes were placed such that their centers were at least 10 cm away from the common anode electrode (Fig. 1B). Motor-level NMES was applied to produce palpable contractions of the trunk muscles (Elgueta-Cancino et al. 2019). The stimulation intensity was adjusted to produce palpable contractions of the left and right sides ES muscles by gradually increasing the stimulating pulse amplitude in 1 mA increments until symmetric and bilateral contractions were generated, as previously described during trunk NMES (Milosevic et al. 2015b; Patel et al. 2017; Elgueta-Cancino et al. 2019). Specifically, at the start of the experiment, the stimulation intensity was set to be just above the level sufficient to produce symmetric contraction of both sides of the ES muscles (Elgueta-Cancino et al. 2019). During the experiments, if the stimulation was deemed insufficient to maintain palpable contraction levels, NMES intensity was re-adjusted (typically increased) until the contraction level was again sufficient to compensate for the effect of muscle fatigue, consistent with a previous trunk NMES study (Elgueta-Cancino et al. 2019). The stimulation amplitudes ranged between 20 and 39 mA, with the final settings of 28.5 ± 3.3 mA for the MEP session and 28.9 ± 5.6 mA for the CMEP session (mean ± SD).

### Data acquisition

Electromyographic (EMG) activity was recorded from the ES muscle unilaterally. Bipolar Ag/AgCl surface electrodes (Vitrode F-150S, Nihon Koden, Tokyo, Japan) were placed, with 1 cm separation, on the left ES muscle on the 12th thoracic vertebral level, 3 cm left side of the spinous processes (Sasaki et al. 2020) (Fig. 1B). A ground electrode was placed over the right anterior superior iliac spine. Prior to the application of electrodes, the skin was cleaned using alcohol swabs to reduce skin impedance. Signals were band-pass filtered (5–1000 Hz) and amplified (× 1000) using a multi-channel EMG amplifier (MEG-6108, Nihon Kohden, Tokyo, Japan). All data were digitized at a sampling frequency of 4000 Hz using an analog-to-digital converter (PowerLab/16SP, AD Instruments, Castle Hill, Australia) and stored on a computer for post-processing.

### Motor evoked potentials (MEPs)

To examine how NMES affects the corticospinal excitability of the trunk muscles, TMS was applied over the primary motor cortex using a mono-phasic magnetic stimulator (Magstim 200, Magstim Co., Whitland, UK) through a double cone coil (outside diameter of 110 mm; Magstim Co., Whitland, UK). Specifically, the optimal stimulation “hot spot” was searched over the contralateral (right) motor cortical area to evoke MEPs from the left ES muscle (Rossini et al. 2015). Prior to the experiments, the motor threshold during upright sitting was set to elicit MEP responses greater than 50 µV in at least five of ten successive trials (Rossini et al. 2015). The TMS intensity for the experiment was then set at 120% of the motor threshold during upright sitting, which was 66.2 ± 13.6% of maximal stimulator output (mean ± SD). The average MEP amplitude across participants at the baseline assessment was 0.11 ± 0.05 mV (mean ± SD). During TMS assessments, the coil position and orientation were monitored throughout the experiment using a tracking system (Brainsight, Rogue Research, Montreal, Canada) (Rossini et al. 2015).

### Cervicomedullary motor evoked potentials (CMEPs)

To examine how NMES affects subcortical excitability of the trunk muscles, CMS was applied on the cervicomedullary junction using a mono-phasic magnetic stimulator (Magstim 200, Magstim Co., Whitland, UK) through a circular coil (diameter of 90 mm; Magstim Co., Whitland, UK), which was placed ipsilateral to the target ES muscle over the left side of the neck below the inion, and with the current flowing downward in the coil (Taylor and Gandevia 2004; Chiou et al. 2018a). The optimal coil position was marked on the head and the coil was held firmly throughout the experiment. Since the ES muscle at the T12 vertebral level is innervated by the dorsal rami of thoracic and lumbar spinal nerves (T8-L3), responses elicited by cervicomedullary stimulation may reflect direct corticospinal activation below the level of the motor cortex (Taylor and Gandevia 2004; Chiou et al. 2018a). Prior to the experiments, while participants remained seated on a chair, CMS intensity was set to elicit rest-level CMEP responses with an amplitude of approximately 0.1 mV (Chiou et al. 2018a, b; Sasaki et al. 2020), which required 69.6 ± 16.7% of maximal stimulator output (mean ± SD). The average CMEP amplitude across participants at the baseline assessment was 0.13 ± 0.05 mV (mean ± SD), which matched MEP amplitudes in the TMS session. Moreover, latencies of CMEP responses were visually determined for each trial from the rectified EMG traces. The average CMEP latencies across participants at baseline assessment was 10.6 ± 2.40 ms (mean ± SD), which is consistent with the previous studies assessing CMEP in the erector spinae muscles (Chiou et al. 2018a, b).

### Data analysis

The peak-to-peak amplitudes of MEP and CMEP responses were evaluated. Ten trials were averaged in the Pre and Post assessments phase. To quantify the responses over the 20 min NMES intervention, the During assessments phase was divided into 5 intervals, each lasting 4 min and consisting of 6 ON / OFF sets (40 s/set × 6 sets = 4 min), which resulted in 18 MEP or CMEP trials (3 trials/set × 6 sets = 18 trials) that were averaged for: During1 (0–4 min); During2 (4–8 min); During3 (8–12 min); During4 (12–16 min); and During5 (16–20 min) intervals. The averaged MEP and CMEP peak-to-peak amplitudes were normalized as a percentage of the baseline (Pre) assessment responses for each participant. Moreover, background EMG activity in a 50 ms window before each MEP or CMEP stimulus was computed using the root mean square of the EMG activity in each trial. Background EMG activities were divided into the same assessment intervals and also normalized as a percentage of the baseline (Pre) for each participant. Finally, latencies of MEP and CMEP responses were visually identified for each trial using rectified EMG traces and grouped in the same assessment intervals for comparison. All calculations were performed using a custom-written script in MATLAB (2017a, The MathWorks Inc., Massachusetts, USA).

### Statistics

Normalized MEP and CMEP peak-to-peak responses, background EMG activities, and latencies were compared between the assessment intervals using the Friedman test, a non-parametric repeated measure one-way analysis of variance (ANOVA). Significant results were followed up with post-hoc multiple comparisons using the Steel test to compare baseline (Pre) to responses during the intervention (During1, During2, During3, During4, During5) as well as after the intervention (Post). Non-parametric tests were chosen because the Shapiro–Wilk test showed that most identified measures were not normally distributed. The significance level was set to p < 0.05. The eta squared values for the Friedman testwere calculated as the effect size indices (Rosenthal et al. 1994; Morse 1999; Cohen 1998). The thresholds for interpreting eta squared values were set at 0.01, 0.06, and 0.14 for small, medium, and large, respectively (Morse 1999; Cohen 1998).

## Results

### MEP responses and background EMG

**Fig. 2:**
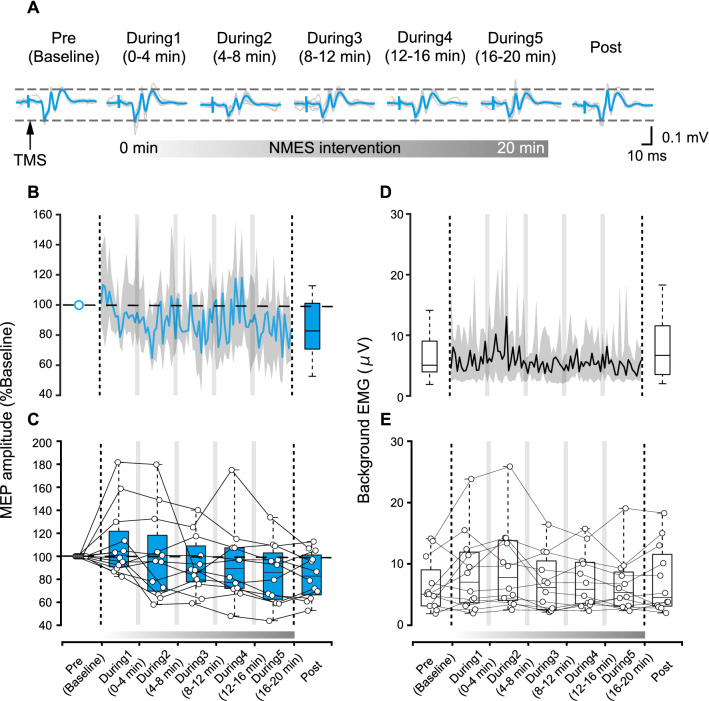
**A** Motor evoked potentials (MEP) in the erector spinae (ES) muscle of a representative participant elicited by transcranial magnetic stimulation (TMS) at baseline (Pre), during the NMES intervention (During 1–5) and after the intervention (Post). Light blue traces indicate averaged MEP responses, while grey traces indicate individual MEP responses. **B** MEP amplitude change at Pre, During, and Post NMES intervention. A line indicates the median across participants and the shaded areas indicate interquartile range. Each MEP in the During assessment phase was collected during NMES OFF periods. **C** Box plots show group data of MEP amplitude during each time window. White dots indicate average data for each individual subject at each time point. **D** Background EMG, calculated in each TMS trial conducted during NMES OFF periods, at Pre, During, and Post NMES intervention. A line indicates the median across participants and the shaded areas indicate interquartile range. Each background EMG in the During assessment phase was obtained from each TMS trial during NMES OFF periods. **E** Box plots show group data of background EMG during each time window. Horizontal lines of the box plots indicate the median values, ends of the boxes represent the 25th and 75th percentiles, while the whiskers illustrate the minimum and maximum values. White dots indicate average data for each individual participant at each time point

Representative ES muscle MEP responses are shown in Fig. 2A, while average results across all participants are shown in Fig. 2B. Since the Friedman test showed a significant effect (χ^2^(6) = 15.85, p = 0.015, η^2^ = 0.29), post-hoc analysis was performed to compare baseline (Pre) to responses during the intervention (During1, During2, During3, During4, During5) and immediately after the intervention (Post). However, the Steel post-hoc test showed no significant differences between the baseline and any time intervals (p > 0.05) (Fig. 2C).

Average background EMG activities across participants are shown in Fig. 2D. The statistical comparison showed that background EMG activities were not significantly different between assessment intervals (χ^2^(6) = 5.71, p = 0.456, η^2^ = 0.08) (Fig. 2E).

The statistical comparison showed that MEP response latencies were not significantly different between assessment intervals (χ^2^(6) = 2.43, p = 0.876, η^2^ = 0.03).

### CMEP responses and background EMG

**Fig. 3:**
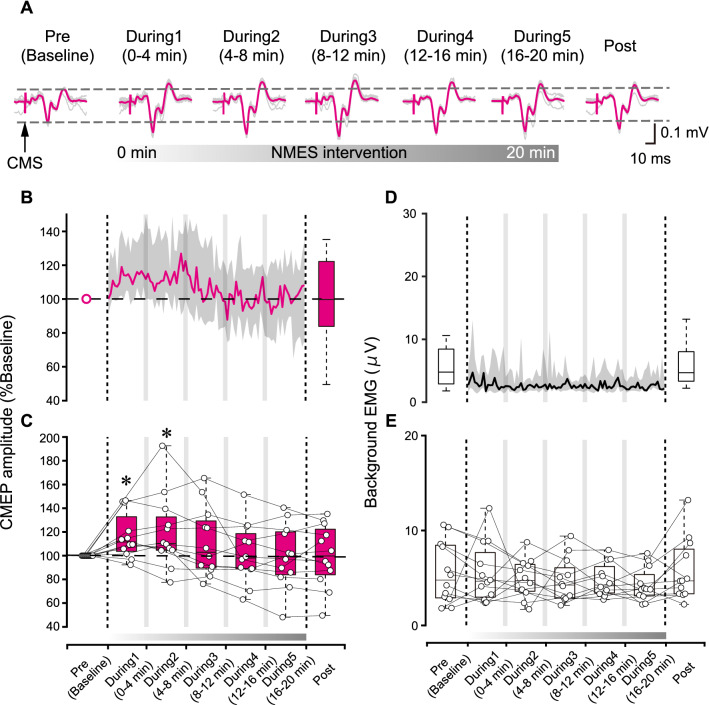
**A** Cervicomedullary motor evoked potentials (CMEP) in the erector spinae (ES) muscle of a representative participant elicited by cervicomedullary junction magnetic stimulation (CMS) at baseline (Pre), during the NMES intervention (During 1–5) and after the intervention (Post). Pink traces indicate averaged CMEP responses, while grey traces indicate individual MEP responses. **B** CMEP amplitude change at Pre, During, and Post NMES intervention. A line indicates the median across participants and the shaded areas indicate interquartile range. Each CMEP in the During assessment phase was collected during NMES OFF periods. **C** Box plots show group data of CMEP amplitude during each time window. White dots indicate average data for each individual subject at each time point. Asterisks indicate significant differences compared to the baseline (Pre). CMEP responses were significantly larger in During1 (p = 0.016) and During2 (p = 0.016) intervals, compared to the baseline (Pre) assessment. **D** Background EMG, calculated in each CMS trial conducted during NMES OFF periods, at Pre, During, and Post NMES intervention. A line indicates the median across participants and the shaded areas indicate interquartile range. Each background EMG in the During assessment phase was obtained from each CMS trial during NMES OFF periods. **E** Box plots show group data of background EMG during each time window. Horizontal lines of the box plots indicate the median values, ends of the boxes represent the 25th and 75th percentiles, while the whiskers illustrate the minimum and maximum values. White dots indicate average data for each individual participant at each time point. Legend: *p < 0.05

Representative ES muscle CMEP responses are shown in Fig. 3A, while average results across all participants are shown in Fig. 3B. Since the Friedman test showed a significant effect (χ^2^(6) = 21.07, p = 0.002, η^2^ = 0.29), post-hoc analysis was performed to compare baseline (Pre) to responses during the intervention (During1, During2, During3, During4, During5) and immediately after the intervention (Post). Specifically, the Steel post-hoc analysis showed that the responses were significantly larger in During1 (p = 0.016, median difference from the baseline: + 11.6%, 95%CI: 3.53 to 29.6%) and During2 (p = 0.016, median difference from the baseline: + 10.3%, 95%CI: 0.03 to 39.8%) intervals, compared to the baseline (Pre) assessment (Fig. 3C). It should be noted that the majority of participants (10 of 12) showed larger CMEP responses in During1 and During2 assessments intervals, compared to the baseline (Pre) assessment (Fig. 3C).

Average background EMG activities across participants are shown in Fig. 3D. The statistical comparison showed that background EMG activities were not significantly different between assessment intervals (χ^2^(6) = 4.429, p = 0.619, η^2^ = 0.06) (Fig. 3E).

The statistical comparison showed that CMEP response latencies were not significantly different between assessment intervals (χ^2^(6) = 6.36, p = 0.384, η^2^ = 0.09).

## Discussion

We investigated how 20 min trunk NMES intervention affects the neural motor circuits by evaluating MEP and CMEP responses during and after the intervention. It was previously demonstrated that both MEP responses (Rothwell et al. 1991) as well as CMEP responses (Ugawa et al. 1994; Taylor et al. 2002) are facilitated during voluntary muscle contractions. Background EMG activities remained the same throughout the assessment intervals in our current study (Figs. 2E and 3E). Therefore, it can be considered that the responses were predominantly affected by the NMES interventions. However, we cannot rule out a possibility that voluntary activations were also present during NMES delivery and that they may have contributed to neuromodulation, warranting future investigations to test trunk NMES in fully relaxed muscles (e.g., prone position). Specifically, our results showed that MEP responses of the trunk muscles were not facilitated by trunk NMES during the intervention, while CMEP responses were facilitated within the first 8 min of applying NMES and returned to baseline before the end of the 20 min intervention period (Figs. 2 and 3). Moreover, MEP responses may have decreased after the intervention. Overall, these results demonstrated that trunk NMES could facilitate CMEP responses but not MEP responses in able-bodied participants. A discussion about the possible neuromodulation mechanisms follows.

### Origins of MEP and CMEP responses of the trunk muscles

The MEP responses of the erector spinae muscle reflect the excitability of the corticospinal tract (Chiou et al. 2018b; Sasaki et al. 2021). Since our method for eliciting trunk muscle MEPs is consistent with previous studies (Chiou et al. 2018b; Sasaki et al. 2021), MEP responses elicited in our current study are also assumed to reflect the excitability of the corticospinal tract.

Moreover, our method for eliciting CMEP responses in the erector spinae muscle using cervicomedullary junction magnetic stimulation delivered through a circular TMS coil is also consistent with previous studies (Chiou et al. 2018a, b). Specifically, the latencies of approximately 10 ms and facilitated responses during voluntary contractions, were previously used to confirm that the elicited CMEPs originated from the stimulation of the corticospinal tract axons, and not the cervical roots and/or the peripheral nerves (i.e., M-wave) (Chiou et al. 2018a, b). Our current study protocol followed the same coil placement/orientation and demonstrated that the latency of the elicited responses is consistent with that of previous studies (Chiou et al. 2018a, b), although we did not formally test modulation of the responses during voluntary muscles contractions (NOTE: in preliminary testing, we did confirm that the responses are modulated during voluntary contractions, but not in the main study protocol). Therefore, CMEP responses elicited in our current study are assumed to originate from the stimulation of the corticospinal tract axons, therefore reflecting subcortical motor circuit excitability.

### Possible subcortical facilitatory mechanism of trunk NMES

Our findings demonstrated facilitation of CMEP responses during erector spinae NMES (Fig. 3), which is consistent with our hypothesis. CMEP responses elicited by stimulation of the corticospinal tract at the cervicomedullary junction reflect subcortical excitability of the trunk muscles (Taylor and Gandevia 2004; Chiou et al. 2018a) and the efficacy of the corticospinal-motoneuronal synapses (Taylor and Gandevia 2004). Therefore, trunk NMES may also have enhanced the efficacy of corticospinal-motoneuronal synapses, which indicates that transmission occurred at the level where corticospinal synapse on the spinal motoneurons. It was previously reported that electrical stimulation of muscles can activate both Ia afferents and α-motoneuron pathways (Bergquist et al. 2011; Lagerquist et al. 2012). The α-motoneuron pathways are activated not only orthodromically, along the axon towards the muscle, but also antidromically toward the cell body in the spinal cord when muscles are stimulated using NMES (Rushton 2003). Since the antidromic volley and inputs from Ia afferents depolarize the α-motoneuron cell body (Rushton 2003), it is possible that postsynaptic activations induced by NMES could attribute to enhancing corticospinal-motoneuronal synaptic transmission, which is reflected by facilitation of CMEP responses. However, the detailed mechanisms of how depolarization of the motoneurons through the combination of Ia input and antidromic activation is postulated to increase corticospinal-motoneuronal synaptic strength is unclear and requires further study. Nonetheless, our study indicates that short-duration trunk NMES affected subcortical motor circuits.

A previous report suggested that CMEP responses were not modulated during upper-limb NMES application (Kaelin-Lang et al. 2002). However, neural control mechanisms of upper-limb and trunk muscles and/or the stimulation methods could explain the differences between these studies, as we discuss later in the next section. Other recent studies also showed that short-duration lower-limb NMES application can inhibit (Jimenez et al. 2018; Milosevic et al. 2019) or facilitate (Kitago et al. 2004) spinal reflex excitability (H-reflex or posterior-root spinal reflex responses). Specifically, continuous stimulation produced inhibition of spinal excitability (Jimenez et al. 2018; Milosevic et al. 2019), while intermittent (i.e., ON/OFF) stimulation protocols produced facilitation of spinal excitability (Kitago et al. 2004), consistent with our results. Until recently, it was thought that assessments of spinal networks of the human trunk are difficult since peripheral nerves innervating the trunk muscles are short and difficult to access from the skin surface. Therefore, methods for spinal circuit assessment such as the H-reflex are unfeasible (Elgueta-Cancino et al. 2019). Our results provided new insights showing that trunk muscle CMEP elicited by CMS can be facilitated using a short-duration application of intermittent NMES.

It should be noted that neuromodulation effects in our study did not last after the intervention period, possibly due to fatigue (i.e., effects only persisted for 8 min during delivery of NMES) (Fig. 2). It was previously shown that NMES could affect the central nervous system anywhere between 15 and 60 min after the stimulating period (Knash et al. 2003; Khaslavskaia and Sinkjaer 2005; Thompson et al. 2011; Milosevic et al. 2019). These effects may depend on the duration of the stimulation intervention. Specifically, delivery of NMES for 20 and 40 min on the limbs was shown to affect the central nervous system excitability, while 60 min was ineffective (Andrews et al. 2013). This may possibly be due to muscle fatigue, which could be induced during NMES (Bergquist et al. 2011). Although it is known that trunk muscles are postural muscles, which are more resistant to fatigue, NMES application over the muscle belly may still be prone to rapid fatigue as the motor unit recruitment order is different compared to that during voluntary contraction of muscles (Bergquist et al. 2011). Therefore, it is possible that NMES could induce muscle fatigue more quickly than voluntary muscles contraction, even if the muscles are resistant to fatigue. It was also reported that amplitude of CMEP responses can be inhibited during fatiguing tasks (Butler et al. 2003; Taylor 2006). Therefore, it cannot be ruled out that both NMES-induced neuromodulation (CMEP facilitation) and fatigue may have occurred within the 20 min intervention, which affected our results in the second half of the intervention. Future studies should therefore attempt to minimize possible fatiguing effects, while maximizing neuromodulation effects during trunk NMES. For instance, it is possible that shorter interventions (e.g., 8 min) could minimize fatigue while simultaneously eliciting neurophysiological changes during trunk NMES. Other methods such as spatially distributed stimulation may also help with fatigue (Sayenko et al. 2014; Barss et al. 2021), although the efficacy of these methods for eliciting changes in the central nervous system has not yet been tested.

### Lack of changes in the corticospinal circuits during trunk NMES

Our study also found that MEP responses were not affected by the NMES intervention despite a significant effect on the Friedman test and a decreasing trend that was observed after the intervention (Fig. 2B). Lack of changes in the MEP responses, with simultaneous CMEP response facilitation, was also shown recently by Benavides and colleagues (Benavides et al. 2020). It should be noted that they used transcutaneous spinal cord stimulation as a method for neuromodulation of upper-limbs in able-bodied participants as well as individuals with spinal cord injury (Benavides et al. 2020) and performed neurophysiological assessment before and after the intervention, while our current study used trunk NMES in able-bodied individuals and we performed assessments before, during and after the intervention. Although the stimulating parameters are considerably different between these techniques (Benavides et al. 2020 used 30 Hz pulses with a 5 kHz carrier frequency), both modes of stimulation aim to provide sensory inputs to the spinal cord via afferent fiber activation. A possible explanation for the lack of changes in corticospinal excitability is that inhibitory cortical circuits projecting onto corticospinal neurons may be involved. Specifically, animal model studies have demonstrated that peripheral afferent input could affect motor cortical activity via dense intracortical projections between the primary motor cortex and the somatosensory cortex (Goldring et al. 1970). Indeed, direct input from the somatosensory cortex can produce both excitation and inhibition in motor cortical cells (Porter et al. 1990). In humans, Benavides and colleagues (Benavides et al. 2020) showed that afferent (dorsal roots) activation by transcutaneous spinal cord stimulation can increase short-interval intracortical inhibition, without changes in MEP responses and simultaneous facilitation of CMEP responses only after the 30 Hz intervention applied with a 5 kHz carrier frequency (Benavides et al. 2020). Although we did not measure intracortical inhibition in the current study, it is possible that NMES also activated inhibitory cortical networks (Bergquist et al. 2011; Milosevic et al. 2020; Carson and Buick 2021), which resulted in the lack of changes in corticospinal excitability. On the other hand, the study by Elgueta-Cancino and colleagues reported that intracortical circuits may not be modulated after application of trunk NMES (Elgueta-Cancino et al. 2019). However, Elgueta-Cancino and colleagues conducted neurophysiological assessments only after the 20 min NMES intervention, while changes during the intervention were not investigated (Elgueta-Cancino et al. 2019). Moreover, neuromodulatory effect of NMES may depend on the stimulation parameter including the frequency and intensity of stimulation, as well as the method of application of NMES (Bergquist et al. 2011; Carson and Buick 2021). Therefore, future studies are warranted to confirm intracortical inhibition during and after trunk NMES while simultaneously monitoring spinal and corticospinal excitability.

Previous studies have reported that application of NMES can either facilitate (Khaslavskaia et al. 2002; Thompson and Stein 2004; Ridding et al. 2005; Barsi et al. 2008) or not affect corticospinal excitability (Kitago et al. 2004), while spinal excitability could either be facilitated (Kitago et al. 2004) or inhibited (Milosevic et al. 2019) after the intervention. Although different effects between studies may be related to stimulation parameters, it is possible that NMES neuromodulatory effects may also depend on the neural control mechanisms and the functional role of investigated muscles. It is well known that trunk muscles have a richer projection from the subcortical circuit, such as the brainstem, the vestibular system, and the spinal cord, compared to the motor cortex (Cottingham et al. 1988; Zedka et al. 2004; Galea et al. 2010). In other words, trunk muscles do not have strong cortical connections, while upper-limb muscles for instance have a much larger somatotopic representation within the primary motor cortex (Penfield and Boldrey 1937). Upper-limb muscles have larger cortical contributions due to their functional role in fine motor control (Palmer and Ashby 1992; Baldissera and Cavallari 1993; Noordhout et al. 1999). This may explain the greater potential for corticospinal neuromodulation in the upper-limb muscles after the application of NMES. Similarly, it was shown that NMES can have different effects on cortical and spinal circuits depending on which lower-limb muscles were stimulated. For instance, stimulation of the soleus was shown not to affect corticospinal excitability (Kitago et al. 2004), while spinal reflex responses could be modulated after short-duration application of NMES (Kitago et al. 2004; Jimenez et al. 2018; Milosevic et al. 2019). In contrast, stimulation of the tibialis anterior does not always affect spinal excitability (Thompson et al. 2011; Obata et al. 2015), while corticospinal excitability could be facilitated after tibialis anterior NMES (Khaslavskaia et al. 2002; Thompson and Stein 2004). Differences in cortical and spinal contributions to the control of soleus and tibialis anterior muscles may explain these results (Morita et al. 2000). Specifically, the soleus has greater spinal connections, while the tibialis anterior is known to have stronger corticospinal inputs (Morita et al. 2000; Lagerquist et al. 2012). Therefore, different muscles may be affected by NMES to a different extent depending on their neural control mechanism and/or their functional role. Our results nevertheless showed that short-duration application of NMES on the trunk extensor muscles did not affect MEP responses during and after the intervention, consistent with our hypothesis and the results of Elgueta-Cancino and colleagues (Elgueta-Cancino et al. 2019), while CMEP responses were facilitated during the intervention period.

### Limitations

Our work has several limitations that should be acknowledged. First, in the current study, we did not quantify intracortical activity using paired-pulse TMS to confirm intracortical inhibition during NMES. By assessing MEP and CMEP excitability during the interventions, we could infer how NMES affects corticospinal networks. Nonetheless, further studies are warranted to specifically test intracortical mechanisms during NMES. Second, NMES was applied bilaterally in our current study, while CMEP and MEP responses were elicited unilaterally from the left side of the trunk muscles. In preliminary testing, we confirmed that unilateral trunk contractions would be unnatural and could possibly generate compensatory movements. Therefore, we chose to apply NMES bilaterally, while evaluating unilateral responses with the assumption that effects would be symmetrical. Third, assessments during the intervention in our study were performed in brief periods when NMES was OFF since stimulating artifacts during NMES ON periods prevented eliciting MEP and CMEP responses reliably. Although this may have limited assessments of responses during NMES-induced muscle contractions, similar protocols have been used in the past to successfully examine neuromodulation effects during NMES interventions (Mang et al. 2010). Fourth, the NMES intervention and neurophysiological assessments were performed while participants were seated on a chair and instructed to maintain an upright unsupported posture, consistent with previous studies investigating CMEP and MEP responses in the erector spinae muscles (Sasaki et al. 2020). However, it is possible that the participant’s posture could affect the intervention efficiency and/or the neurophysiological assessments due to back and abdominal muscle background activations. Therefore, future studies should consider applying trunk NMES during the prone posture when the muscles are completely relaxed to further understand the effects of trunk NMES on the central nervous system excitability. Fifth, it is also possible that the responses elicited in our current study were at least partly due to cervical root and/or peripheral nerve activation, although evidence suggests that CMEP responses reflect excitability of subcortical motor circuits in the corticospinal tract (see section Origins of MEP and CMEP responses of the trunk muscles). Sixth, we didn't have control conditions where participants were instructed to remain relaxed and seated without the application of NMES. Therefore, it is possible that the CMEP may have been influenced by rest. Finally, we showed that trunk NMES could facilitate CMEP responses by approximately 10%. While these findings are statistically significant, the functional and/or physiological significance of this facilitation has not been tested. Therefore, future studies should examine whether NMES-induced increases in CMEP responses contribute to postural control performance not only in able-bodied subjects but also in patient populations to establish practical applications of the approach shown in our current study.

## Data Availability

Data presented in this manuscript were newly collected for this study. Due to data privacy issues, they are unavailable to the community in open repositories. The datasets generated in this study are available from the corresponding author upon reasonable request. Considerations will be given based on the review of reasons for requesting the data and the procedures to ensure data privacy.
